# Analytical performance evaluation of Lumipulse® SARS‐CoV‐2 antigen assay in 392 asymptomatic patients

**DOI:** 10.1002/jcla.24867

**Published:** 2023-03-27

**Authors:** Arnolfo Petruzziello, Rocco Sabatino, Livia Anna Catapane, Carmela De Falco, Antonella Petti, Elena Tripaldelli, Giovanna Loquercio, Angela Annecchiarico, Angela Salzillo, Eugenio Caradonna, Paolo Maggi

**Affiliations:** ^1^ UOC Patologia Clinica, Dipartimento dei Servizi Sanitari AORN S.Anna e S.Sebastiano Caserta Italy; ^2^ Direzione Sanitaria AORN S.Anna e S.Sebastiano Caserta Italy; ^3^ UOC Malattie infettive e tropicali, Dipartimento Scienze Mediche AORN S.Anna e S.Sebastiano Caserta Italy; ^4^ Unità di Ricerca Gemelli Molise Campobasso Italy; ^5^ Dipartimento di Malattie Infettive Università della Campania Luigi Vanvitelli Caserta Italy

**Keywords:** asymptomatic, comparison, COVID‐19, RT‐PCR, SARS‐CoV‐2 antigen

## Abstract

**Introduction:**

Severe acute respiratory syndrome coronavirus 2 (SARS‐CoV‐2) infection is one of the current public health care challenges. The main strategy adopted to prevent the spread of infection is the rapid identification of COVID‐19‐positive subjects. The aim of this study was to compare the performance of Lumipulse® antigen immunoassay with the real‐time RT‐PCR, the gold standard for the diagnosis of SARS‐CoV‐2 infection, in a strictly selected asymptomatic population.

**Materials and Methods:**

A total of 392 consecutive oro‐nasopharyngeal swabs were collected from patients with no symptoms related to COVID‐19 at the Emergency Department of AORN Sant'Anna e San Sebastiano, Caserta, Italy to evaluate the analytical performance of Lumipulse® SARS‐CoV‐2 antigen compared to qualitative real‐time RT‐PCR in asymptomatic patients.

**Results:**

Lumipulse® SARS‐CoV‐2 antigen assay shows an overall agreement rate of 97% with a sensitivity of 96% and a specificity of 98%, with a PPV and NPV of 97%. The sensitivity varies according to the cycle threshold (*C*_t_)‐value reaching 100% and 86% with 15 < *C*_t_ < 25 and *C*_t_ ≥ 25, respectively. The ROC analysis yielded an AUC value of 0.98, suggesting that the antigen test may accurately detect SARS‐CoV‐2.

**Conclusion:**

Our data showed that Lumipulse® SARS‐CoV‐2 antigen assay might be an efficient tool in the identification and limitation of SARS‐CoV‐2 transmission in large asymptomatic populations.

## Graphical abstract

The study evaluates the performance of the Lumipulse® SARS‐CoV‐2 antigen assay able to detect SARS‐CoV‐2 nucleocapsid antigen in oro‐nasopharyngeal swab samples by comparing it with qualitative real‐time RT‐PCR. The data obtained show that SARS‐CoV‐2 antigen assay might be an efficient tool in the identification and limitation of SARS‐CoV‐2 transmission.

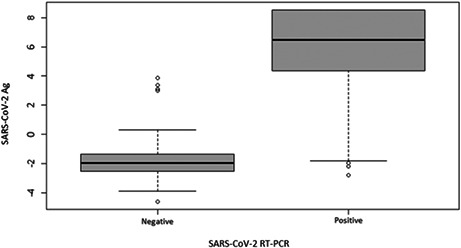

## 1 INTRODUCTION

Severe acute respiratory syndrome coronavirus 2 (SARS‐CoV‐2) is the etiological agent of coronavirus disease 2019 (COVID‐19), firstly described in Wuhan, China in December 2019^1^ and after its rapid diffusion, characterized by a high number of cases worldwide, it has been classified in March 2020 by the World Health Organization (WHO) as a pandemic.^2^ Globally, since the beginning of the pandemic, WHO has reported approximately 590,000,000 cases of SARS‐CoV‐2 infection, with over 6,500,000 deaths, and in Italy, about 21,500,000 cases and more than 170,000 deceases.^3^, ^4^

SARS‐CoV‐2 is a Coronavirus with a diameter of 80–120 nm, belonging to the order of Nidovirales, with a positive‐sense single‐stranded RNA genome sized from 26 to 32 kilobases which codify four main structural proteins: spike (S), nucleocapsid (N), membrane (M), and envelope (E).^5^, ^6^, ^7^ The binding of the Spike protein to the angiotensin‐converting enzyme 2 (ACE‐2) receptor, widely present in epithelial cells, is the first step in SARS‐CoV‐2 infection.^8^, ^9^, ^10^

After 2 years of the pandemic, it is now widely clear that the most useful tool for controlling the spread of SARS‐Cov2 infection is the rapid identification of potentially infected individuals, may be the only strategic policy able to prevent the outbreak of new pandemic waves. Among the different analytical methods nowadays commercially available, the use of last‐generation antigenic tests has become recently more and more attractive, especially considering their rapidity and low costs, even though the gold standard still remains the molecular diagnosis characterized by a high sensitivity and specificity (>95%).^11^, ^12^, ^13^, ^14^

As recently reported, the SARS‐CoV‐2 nucleocapsid protein (NP) is an ideal target for a viral antigen‐based diagnosis, especially considering its high immunogenicity and its massive expression during the early phases of the infection.^7^, ^9^, ^10^, ^11^, ^12^, ^13^, ^14^, ^15^

The aim of this study has been to evaluate the performance of Lumipulse® SARS‐CoV‐2 antigen test (Fujirebio, Inc.), an automated quantitative chemiluminescence enzyme immunoassay technology (CLEIA) used to detect SARS‐CoV‐2 NP in oro‐nasopharyngeal swab samples of asymptomatic patients, and compare its performance with qualitative real‐time RT‐PCR as reference test, with the purpose to demonstrate how this strategy can be useful for the early detection of new cases in order to prevent new pandemic waves.

## 2 MATERIALS AND METHODS

### 2.1 Samples collection

A total of 392 consecutive oro‐nasopharyngeal swabs from patients referred to the Emergency Department of AORN Sant'Anna e San Sebastiano, Caserta, Italy, for various clinical conditions (25% arrhythmias or cardiac injury, 15% renal disorders, 21% polytrauma, 12% neurological symptoms, and 27% other pathologies) and without any of the clinical symptoms generally related to COVID‐19 (cough, fever, myalgia, headache, dyspnea, etc.) were collected from October to December 2020. The median age was 59.6 years (range 1–101). Male patients were 53% (*n* = 208) and females were 47% (*n* = 184) and over 70% of them were older than 50 years (Table 1). Samples were obtained using cotton swabs and viral transport media in UTM1 (Copan Diagnostics). Specimens were collected at the time of admission and stored at +4°C until nucleic acid extraction. SARS‐CoV‐2 antigen test was performed within 2 h after swab collecting and within 24 h the results were confirmed by real‐time RT‐PCR assay.

**TABLE 1:** Patient characteristics of the study population.

	Number of patients (%)
Gender	
Male	208 (53.1)
Female	184 (46.9)
Age	
≤50 years	115 (29.3)
>50 years	277 (70.7)

All procedures followed were in accordance with the ethical standards and the study complies with the Declaration of Helsinki. Since all the diagnostic activities described in this study were part of routine laboratory operations necessary for SARS‐CoV‐2 screening and diagnosis at the local facility, no patient informed consent and Ethical Committee were necessary. The authors were not involved in the sample collection and they had no access to patient identifying information.

### 2.2 SARS‐CoV‐2 antigen detection assay

A quantity of 500 μL of viral transport media from each oro‐nasopharyngeal swab was centrifuged at 3000 *g* for 10 min. The antigen level was measured with the Lumipulse SARS‐CoV‐2 Ag kit (Fujirebio, Inc.) on the Lumipulse G600II automated immunoassay analyzer (Fujirebio, Inc.), following the Manufacturer's instructions. The samples were incubated with the anti‐SARS‐CoV‐2 Ag monoclonal antibody‐coated magnetic particle solution and after 10 min at 37°C the alkaline phosphatase‐conjugated anti‐SARS‐CoV‐2 Ag monoclonal antibody was added and incubated for other 10 min at 37°C. In the end, the substrate solution was added and incubated for 5 min at 37°C. The resulting reaction signals were proportional to the amount of SARS‐CoV‐2 Ag in the sample, allowing a quantitative determination for SARS‐CoV‐2 Ag in the oro‐nasopharyngeal swabs. The negative cut‐off was <1.64 pg/mL and the positive one was >10 pg/mL; values between 1.64 and 10 were considered as a grey zone.

### 2.3 Viral RNA extraction

PANA 9600s automated extraction platform (Xi'an Tianlong Science and Technology Co., LTD) was used to extract SARS‐CoV‐2 RNAs from 200 μL of oro‐nasopharyngeal swabs using Viral DNA and RNA extraction Kit. Extraction was performed according to the manufacturer's instructions. Briefly, the magnetic beads that have been bound to nucleic acids are magnetized, transferred, and released via the specialized magnetic rods during the process of extraction. Viral RNA was eluted with 20 μL buffer and used for RT‐PCR assay.

### 2.4 SARS‐CoV‐2 RNA detection using real‐time RT‐PCR

The novel Coronavirus (2019‐nCoV) Nucleic Acid Detection Kit (Suzhou TianLong Biotechnology), which targets ORF1ab and nucleocapsid (N) genes of SARS‐CoV‐2, was used for SARS‐CoV‐2 RNA detection according to the manufacturer's instructions. Briefly, the mix solution was composed of 5 μL of extracted RNA, 17 μL of the reaction solution, 1.5 μL of enzyme mix, and 1.5 μL of primer and probe. The Gentier 96 real‐time thermal cycler (Tianlong, China) was used for amplification. The conditions consisted of 1 cycle of 30 min at 50°C, 1 min at 95°C, 5 cycles of 30 s at 95°C, 10 s at 50°C, and 10 s at 72°C followed by 35 cycles of 3 s at 95°C and 30 s at 58°C. The results were analyzed using Gentier Real‐time PCR system (Xi'an Tianlong Science and Technology Co., LTD), and a cycle threshold value (*C*_t_‐value) < 35 for at least one target gene was defined as a positive result.

### 2.5 Statistical analysis

Descriptive statistics were used to describe general information of patients. Continuous data were presented as mean, standard deviation (SD), median and interquartile range (lower and upper quarter). Categorical data were presented in numbers, percentages, and a 95% confidence interval (95% CI). A comparison of non‐parametric data between results from SARS‐Cov2 Ag test and those from SARS‐CoV2 PCR test (gold standard) was done using Mann–Whitney *U*‐test. A *p*‐value of <0.05 was considered statistically significant. Receiver operating characteristics (ROC) curve analysis was used to calculate the area under the curve (AUC), which represents the probability that the test can discriminate between healthy and disease states. The AUC values range from 0 to 1, with an AUC of 1 indicating 100% probability that a given test can discriminate between healthy and SARS‐CoV‐2 infection. The AUC, sensitivity, specificity, negative predictive value (NPV), positive predictive value (PPV), +LR and −LR, diagnostic odds ratio (DOR), and the corresponding 95% CI were calculated for Lumipulse® SARS‐CoV‐2 antigen assay. All analyses were performed with R studio software (version 3.5.3; R studio).

## 3 RESULTS

Of the 392 patients included in the study and tested using Lumipulse® SARS‐CoV‐2 assay, 36.7% (*n* = 144) were positive, while 59.2% (*n* = 232) were negative. In accordance with the manufacturer's instructions, 16 samples (4.1%) were considered as in grey zone (range 1.67–9.19 pg/mL), as shown in Table 2.

**TABLE 2:** Results of SARS‐CoV‐2 Ag test in the selected cohort of 392 nasopharyngeal swabs.

Lumipulse SARS‐CoV‐2 Ag	Results *n* (%)	Male *n* (%)	Median age (years)	Female *n* (%)	Median age (years)
Positive	144 (36.7)	86 (59.7)	67	58 (40.3)	41
Negative	232 (59.2)	113 (48.7)	68	119 (51.3)	67
Grey zone	16 (4.1)	9 (56.2)	61	7 (43.8)	72

Of the 144 NP‐positive patients, 59.7% (*n* = 86) were male with a median age of 67 years old (range 1–101) and 40.3% (*n* = 58) were female with a median age of 41 years old (range 5–92) while of the 232 NP‐negative samples, 48.7% (*n* = 113) were male with a median age of 68 years old (range 18–91) and 51.3% (*n* = 119) were female with a median age of 60 years old (range 2–91), as shown in Table 2. Any significant difference was found between positivity and gender.

We then divided the studied population into two groups based on age (≤50 and >50 years old). Among the NP‐positive samples, 36.8% (*n* = 53) were under 50 years old (34% male and 66% female) and 63.2% (*n* = 91) were older than 50 years old (74.7% male and 25.3% female); instead, among the negative NP samples, 25.4% (*n* = 59) were under 50 years old (32.2% male and 67.8% female) and 74.6% (*n* = 173) were over 50 years old (54.3% male and 45.7% female), as shown in Table 3. Any significant difference was found between positivity and age.

**TABLE 3:** Population studied was divided into two groups based on age (≤50 and >50 years old).

Groups	Male *n* (%)	Female *n* (%)	Total (%)
Positive			
≤50 years	18 (34)	35 (66)	53 (36.8)
>50 years	68 (74.7)	23 (25.3)	91 (63.2)
Negative			
≤50 years	19 (32.2)	40 (67.4)	59 (25.4)
>50 years	94 (54.3)	79 (45.7)	173 (74.6)

The median NP concentration was 688.3 pg/mL (range 11.74–5000 pg/mL) in the group of positive samples and 0.14 pg/mL (range 0.01–1.38 pg/mL) in the group of the negative ones.

Then, we investigated the sensitivity and specificity of Lumipulse® using real‐time RT‐PCR as a reference test. Of the 144 positive antigen samples, 140 were confirmed positive (97.2%) while of the 232 negative antigen samples, 226 were confirmed negative (97.4%). The results showed a sensitivity of 96% (95% CI, 93%–99%) and a specificity of 98% (95% CI, 97%–100%), with PPV of 97% (95% CI, 93%–99%) and an NPV of 97% (95% CI, 94%–99%) with an overall agreement rate of 97% (366/376) as shown in Table 4.

**TABLE 4:** Comparison of RT‐PCR and antigen assay results in the selected cohort of 392 nasopharyngeal swabs.

Lumipulse SARS‐CoV‐2 Ag	Real‐time RT‐PCR		Total
	Positive	Negative	
Positive	140	4	144
Negative	6	226	232
Total	146	230	376
Apparent prevalence	0.38 (0.33–0.43)		
True prevalence	0.39 (0.34–0.44)		
ROC curve's AUC at cut‐off	0.98 (0.96–0.99)		
Sensitivity	0.96 (0.93–0.99)		
Specificity	0.98 (0.97–1.00)		
PPV	0.97 (0.93–0.99)		
NPV	0.97 (0.94–0.99)		
+LR	55.14 (20.86–145.74)		
−LR	0.04 (0.02–0.09)		
DOR	1318.33 (365.59–4753.88)		

*Note*: Sensitivity, specificity, AUC (area under the curve), negative predictive value (NPV), positive predictive value (PPV), positive likelihood ratio (+LR) and negative likelihood ratio (−LR), diagnostic odds ratio (DOR), and the corresponding confidence interval (95% CI) were calculated for Lumipulse® SARS‐CoV‐2 antigen assay using real‐time RT‐PCR as reference.

**FIGURE 1:**
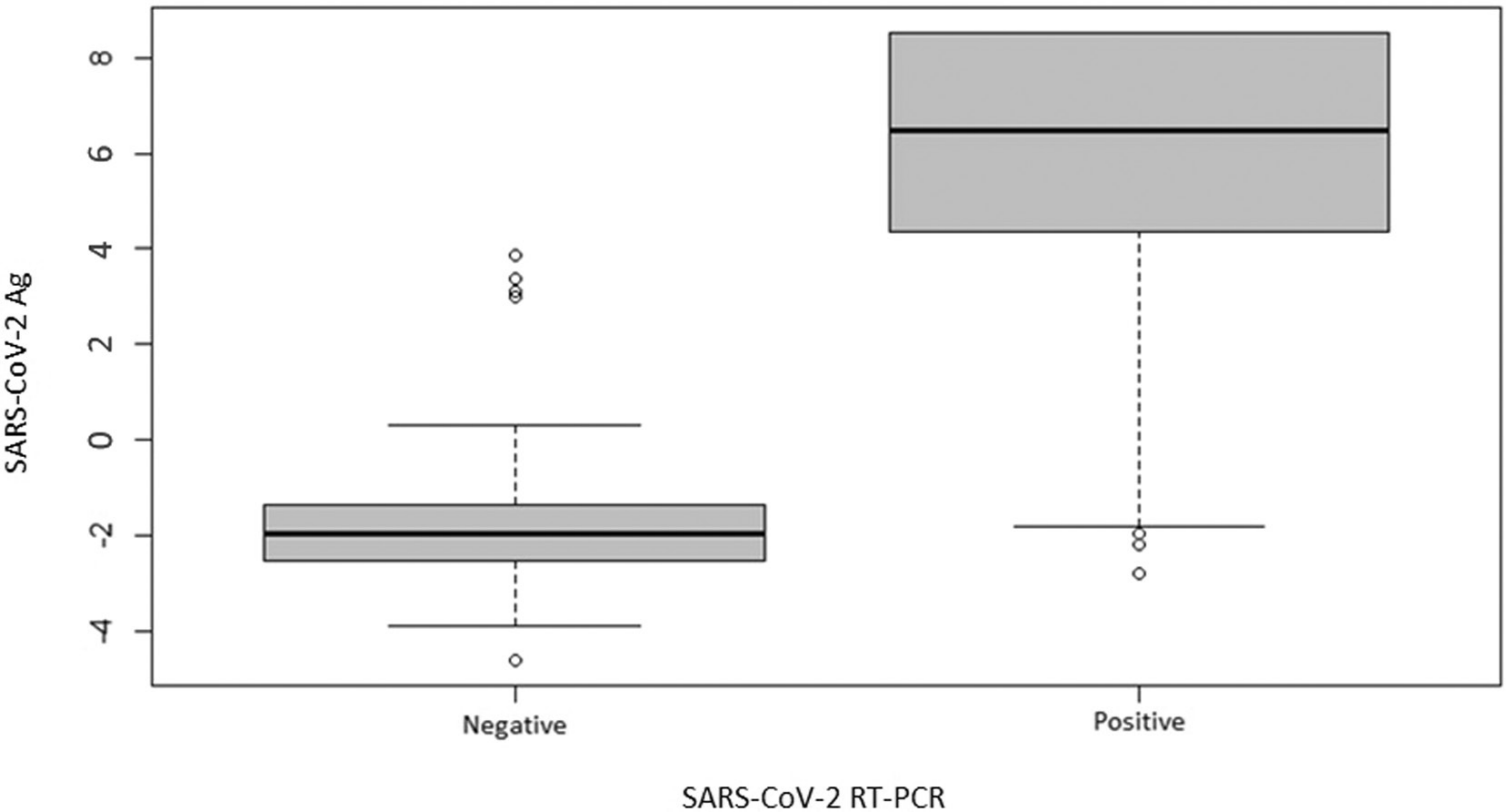
Comparison of the results between the SARS‐CoV‐2 antigen (Ag) test and RT‐PCR. Box plot shows that mean antigen concentration among the PCR‐positive samples was significantly higher than among PCR‐negative samples (*p* < 0.00001, *W* = 720.5).

The mean NP concentration among the PCR‐positive samples was significantly higher than among PCR‐negative samples (*p* < 0.00001, *W* = 720.5) (Figure 1). Moreover, considering the 10 discordant results (DR), we found that six were false negative (FN; three males and three females), and four false positive (FP) (two males and two females), as shown in Table S1 and no one of them was positive for ORF1ab gene. Furthermore, analyzing the mean cycle thresholds (*C*_t_)‐value of the FN samples, we noticed that the mean *C*_t_ was 33.5 ± 1.35 (min 31.6–max 34.8) and exclusively referred to the N gene (median *C*_t_‐values of 34.1). The NP mean concentration of the four FP patients was 29.45 ± 12.22 pg/mL (median value of 25.63 pg/mL).

Ten of the 16 samples (68.7%) considered as grey zone were RT‐PCR negative and six (31.3%) were positive but only for N gene, with a mean *C*_t_ of 27.13 ± 5.29 (Table S2).

**FIGURE 2:**
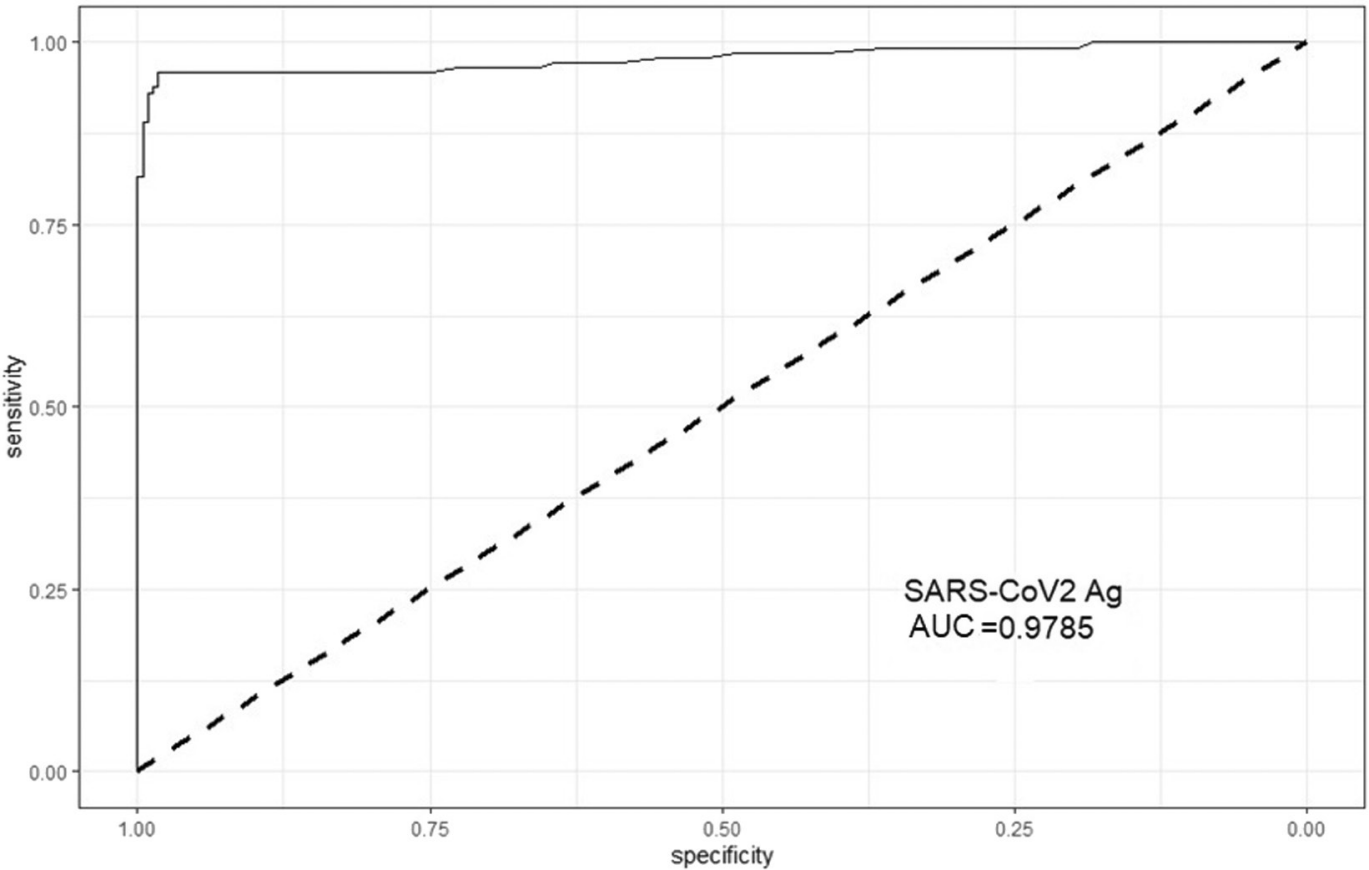
Receiver operating characteristic (ROC) curve analysis. The Ag test achieved an area under the ROC curve (AUC) value of 0.9785 (95% CI: 0.9613–0.9958).

A ROC curve analysis was then performed in order to establish the antigen cut‐off value necessary to establish the SARS‐CoV‐2 infection status identifying an antigen level of 6.56 pg/mL to reach the highest level of accuracy of the test. The ROC analysis yielded an AUC value of 0.9785 (95% CI: 0.96–0.99), suggesting that the antigen test may accurately detect SARS‐CoV‐2 (Figure 2).

Furthermore, in order to compare the performance of the NP antigen test and RT‐PCR test, we divided our population into three groups in accordance with their *C*_t_‐values. In this way, we clearly noticed that in the group with higher *C*_t_ (≥25). the concordance between the antigen test and RT PCR test was lower (86.2%; 95% CI, 73.7%–98.7%), whereas in the second (15 < *C*_t_ < 25) and in the third group (*C*_t_ ≤ 15). concordance was 100%. In addition, in the first group (*C*_t_ ≥ 25), the median concentration of antigen was 46.84 pg/mL (range 0.06–811.94 pg/mL), while in the second (15 < *C*_t_ < 25) and in the third group (*C*_t_ ≤ 15), it was clearly higher (688.3 pg/mL; range 19.90–5000 pg/mL and 4891.45 pg/mL; range 39.6–5000 pg/mL, respectively; Table 5).

**TABLE 5:** Population studied is divided into three groups based on *C*_t_‐value.

Groups	Real‐time RT‐PCR	Lumipulse SARS‐CoV‐2 Ag positive	Sensitivity (%)	Median antigen value (pg/mL)
*C*_t_ ≥ 25	29	25	86	46.84
15 < *C*_t_ < 25	62	62	100	688.3
*C*_t_ ≤ 15	55	55	100	4891.45

We finally divided the group with a higher *C*_t_‐value into two subgroups, 25 < *C*_t_ < 30 and *C*_t_ ≥ 30, and we observed a concordance of 100% and 83.3% (95% CI, 72.1–94.5%), respectively. Furthermore, the median antigen concentration of *C*_t_ ≥ 30 group was 0.25 pg/mL (range 0.06–118.67 pg/mL), while the median antigen concentration of 25 < *C*_t_ < 30 group was 60.29 pg/mL (range 28.15–811.94 pg/mL; Table S3).

## 4 DISCUSSION

Identifying SARS‐CoV‐2‐positive cases among the overall asymptomatic population is still now the more reliable tool to limit the spread of SARS‐CoV‐2 infection. That is the reason for which we chose to focus our study comparing the performance of Lumipulse® antigen assay with real‐time RT‐PCR in a strictly selected COVID‐19 asymptomatic population (no clinical signs and no contacts with confirmed or suspected COVID‐19 cases) particularly difficult to obtain in a pandemic phase characterized by a high viral prevalence (>10%) in order to propose an innovative laboratory methodology as a tangible and reliable solution to reduce the pressure on the public health system during the pandemic phases. Moreover, to our knowledge, this is the first published study in which the performance of a laboratory method for the rapid diagnosis of SARS‐CoV‐2 infection is evaluated in a strictly selected asymptomatic population.

The data obtained in our study show that Lumipulse® has high sensitivity and specificity (96% and 98%, respectively), even if its sensitivity seems to be *C*_t_‐value dependent. Indeed, we noticed a decrease of sensitivity from 100% to 86% as the *C*_t_‐value increases and this is clearly observable in the subgroup with *C*_t_ ≥ 30, in which the sensitivity decreases up to 83%. Our data seem to widely confirm what published by Hirotsu et al. that described that in samples with a viral load of <100 copies, the accuracy of Lumipulse® decreased, showing 100% concordance when the viral load is >100 copies.^15^ Moreover, we found six false‐negative (FN) and four false‐positive (FP) samples. The high median *C*_t_‐values of FN samples could indicate that these patients were probably either in the late phase or in the recovery one, or definitely that the samples were collected in a very early phase of the infection characterized by a low viral load not revealed by the CLIA method. The low percentage of false positive (0.2%) suggests the high reliability of Lumipulse antigen test, although a case report of false‐positive antigen test result was reported in the literature.^16^ It is also interesting to highlight that no one of the samples so‐called Discordant (DR) showed RT‐PCR positivity for the SARS‐CoV‐2 target gene ORF1ab, especially the six false‐negative samples all exclusively characterized by low *C*_t_‐values of N gene. These data might be explained by a phenomenon of “primers and/or probe mismatch”, able to invalidate the RT‐PCR results.

ROC curve analysis performed with the aim to determine the cut‐off value of the Lumipulse test able to establish the higher correlation with SARS‐CoV‐2 infection status showed that when the cut‐off value was set to 6.56 pg/mL, the accuracy reached its highest level (AUC: 0.9785, 95% CI: 0.9613–0.9958). This cut‐off value is highly close to that of 10.0 pg/mL proposed by the manufacturer.

The excellent PPV and NPV estimated in our study seem to encourage the use of Lumipulse® test in many epidemiological scenarios, especially for monitoring asymptomatic large population groups, such as students, sanitary workers, prisoners, etc.^17^ Considering the prevalence of SARS‐CoV‐2 in our area at the time of the study (>10%), our data seem to indicate a last‐generation antigen test might be particularly useful for a policy of large‐scale screening of asymptomatic populations. On the contrary, the use of RT‐PCR still remains mandatory for symptomatic patients, as previously reported.^11^, ^14^, ^17^, ^18^

It is evident that this study has some limitations. First of all, the selected population was composed only of asymptomatic patients excluding those with specific clinical symptoms of SARS‐CoV‐2 infection or other respiratory symptoms. The rationale of this exclusion criteria is strictly related to the aim of the study: to assess the diagnostic performance of the Lumipulse antigen test as screening test in a large and not at‐risk population. So, we cannot exclude that the performance of this test might largely differ in another population with different clinical characteristics.^19^, ^20^, ^21^, ^22^, ^23^ In addition, we compared a quantitative assay as Lumipulse SARS‐CoV‐2 Ag with a qualitative one (real‐time RT‐PCR), but our purpose was only to evaluate the performance of Lumipulse test as a screening test in an asymptomatic population, avoiding any evaluation on its quantitative performance, as previously described.^12^, ^15^, ^19^, ^20^, ^21^, ^22^, ^23^, ^24^

In conclusion, it is evident that the relevance of this study is considered as a potential model for preventing SARS‐CoV‐2 infection in large asymptomatic populations. Indeed, it is now clear that asymptomatic COVID‐19 adult outpatients play a crucial role as transmitters of SARS‐CoV‐2 infection in the hospital environment and therefore their early detection may certainly be an effective strategy to prevent the recrudescence of new pandemic waves. Moreover, considering the more and more reduced resources destined for the public health facilities, the Lumipulse SARS‐CoV‐2 antigen test shows many advantages: easy to use (completely automatized), reduced analysis times (approximately 45 min), and lower operational costs.^17^, ^18^, ^19^, ^20^, ^21^, ^22^, ^23^, ^24^ In addition, its diagnostic performance not seems to be afflicted by the new SARS‐CoV‐2 variants, which recently appeared in the pandemic scenario,^25^, ^26^, ^27^, ^28^ although further studies will be necessary to confirm these data.

## CONFLICT OF INTEREST STATEMENT

No potential conflict of interest was reported by the author(s).

## Supporting information


Appendix S1

## Data Availability

The data that support the findings of this study are available from the corresponding author upon reasonable request.
