# Supplementary material for: Analytical performance evaluation of Lumipulse® SARS‐CoV‐2 antigen assay in 392 asymptomatic patients
Source: J Clin Lab Anal. 2023 Mar 27;37(6):e24867. doi: 10.1002/jcla.24867 (PMC10156099; doi:10.1002/jcla.24867)
Supplement: Supplementary file 1 — Appendix S1 [file JCLA-37-e24867-s001.docx]

| **Gender** | **Age** | **Real time RT-PCR** | | **Lumipulse**  **SARS-CoV-2 Ag** | **Interpretation** |
| --- | --- | --- | --- | --- | --- |
|  |  | ORF1ab | N | (pg/mL) |  |
| F | 43 | TNR | 34,8 | 0,16 | FN |
| M | 90 | TNR | 34,1 | 0,25 | FN |
| M | 53 | TNR | 34,4 | 0,11 | FN |
| F | 33 | TNR | 33.9 | 0,06 | FN |
| M | 60 | TNR | 32,6 | 0,19 | FN |
| F | 78 | TNR | 31,6 | 0,14 | FN |
| M | 69 | TNR | TNR | 28,63 | FP |
| F | 60 | TNR | TNR | 22,63 | FP |
| M | 76 | TNR | TNR | 46,90 | FP |
| F | 34 | TNR | TNR | 19,63 | FP |

Supplementary Table 1. Interpretative table of the discordant results. TNR= target not revealed; FN= False negative; FP= False positive

| **Gender** | **Age** | **Real time RT-PCR** | | **Lumipulse Ag** | **Interpretation** |
| --- | --- | --- | --- | --- | --- |
|  |  | ORF1ab | N | (pg/mL) |  |
| F | 71 | TNR | TNR | 3,96 | Negative |
| M | 20 | TNR | TNR | 5,37 | Negative |
| M | 85 | TNR | TNR | 1,66 | Negative |
| M | 80 | TNR | TNR | 1,65 | Negative |
| F | 72 | TNR | TNR | 1,72 | Negative |
| M | 60 | TNR | TNR | 5,56 | Negative |
| F | 65 | TNR | TNR | 2,2 | Negative |
| M | 54 | TNR | TNR | 1,69 | Negative |
| M | 50 | TNR | TNR | 5,49 | Negative |
| F | 79 | TNR | TNR | 1,86 | Negative |
| F | 75 | TNR | 21,68 | 5,33 | Positive |
| M | 61 | TNR | 35,14 | 2,98 | Positive |
| M | 77 | TNR | 27,29 | 2,44 | Positive |
| F | 72 | TNR | 26,02 | 9,19 | Positive |
| M | 65 | TNR | 31 | 5,44 | Positive |
| F | 62 | TNR | 21,68 | 4,25 | Positive |

Supplementary Table 2. Interpretative table of grey-zone antigen results

| **Groups** | **Real time RT-PCR** | **Lumipulse SARS-CoV-2 Ag positive** | **Sensivity** | **Median antigen value (pg/mL)** |
| --- | --- | --- | --- | --- |
|  |  |  |  |  |
| **25< Ct <30** | 18 | 18 | 100% | 60.29 |
| **Ct ≥ 30** | 11 | 5 | 83% | 0.25 |

Supplementary Table 3. Population studied is divided into two groups based on Ct-value >25
